# Insulin resistance is linked to a specific profile of immune activation in human subjects

**DOI:** 10.1038/s41598-021-91758-3

**Published:** 2021-06-10

**Authors:** Renaud Cezar, Delphine Desigaud, Manuela Pastore, Lucy Kundura, Anne-Marie Dupuy, Chantal Cognot, Thierry Vincent, Christelle Reynes, Robert Sabatier, Elisabeth Maggia, Pierre Corbeau

**Affiliations:** 1 Immunology Department, Nîmes University Hospital, Place du Pr Debré, 30029 Nîmes, France; 2 Institute of Human Genetics, CNRS-Montpellier University UMR9002, 141 rue de la Cardonille, 34396 Montpellier Cedex 5, France; 3 Institute of Functional Genomics UMR5203 and Biocampus UAR3426, CNRS, Inserm, Montpellier University, 141 rue de la Cardonille, 34396 Montpellier, France; 4 Biochemestry Department, Montpellier University Hospital, 371 avenue du Doyen Gaston Giraud, 34295 Montpellier, France; 5 Immunology Department, Montpellier University Hospital, 80 avenue Auguste Fliche, 34295 Montpellier, France; 6 Caisse Primaire D’Assurance Maladie, 14 rue du cirque Romain, Nîmes, France; 7 Montpellier University, 5 Boulevard Henri IV, 34967 Montpellier, France; 8 Fédération Hospitalo-Universitaire Infections Chroniques, Montpellier-Nîmes, France

**Keywords:** Immunology, Inflammation, Chronic inflammation, Endocrinology, Endocrine system and metabolic diseases, Metabolic syndrome, Biochemistry, Glycobiology, Biomarkers

## Abstract

We tested the hypothesis that a particular immune activation profile might be correlated with insulin resistance in a general population. By measuring 43 markers of immune, endothelial, and coagulation activation, we have previously shown that five different immune activation profiles may be distinguished in 150 volunteers. One of these profiles, Profile 2, characterized by CD4+ T cell senescence, inflammation, monocyte, B cell, and endothelial activation, presented elevated insulinemia, glycemia, triglyceridemia, and γ-glutamyl transferase, a marker of liver injury, in comparison with other profiles. Our data are compatible with a model in which a particular immune activation profile might favor the development of insulin resistance and metabolic syndrome. In this hypothesis, identification of this profile, that is feasible with only 3 markers with an error rate of 5%, might allow to personalize the screening and prevention of metabolic syndrome-driven morbidities as liver steatosis.

## Introduction

Metabolic Syndrome (MetS) is a cluster of bioclinical abnormalities including hypertension, central obesity, impaired glucose metabolism, hypertriglyceridemia, and low high-density lipoprotein (HDL) cholesterolemia^1^. These abnormalities are risk factors for developing cardiovascular disease, nonalcoholic fatty liver, type 2 diabetes, neurocognitive disorders, and death from all causes^2^. The prevalence of MetS is on the increase worldwide and affects more than 20% of the global adult population^3^. A root cause of MetS is insulin resistance (IR)^1^. It is therefore of major interest to identify the causes of IR and the biomarkers able to predict its development.

IR is fueled by immune activation (IA). Thus, for instance, the inflammatory cytokines tumor necrosis factor α (TNFα), interleukin (IL)-1β, and IL-6 inhibit insulin signaling^4^. We have recently shown, using 43 cell surface and soluble markers, that distinct IA profiles may be distinguished by a double hierarchical clustering in 150 people aged 55–69 years who were visiting a health center for routine check-up^5^. Moreover, some of these profiles were linked to potential causes of IA as a low level of regulatory T cells, a CD4+ T cell subpopulation able to downregulate IA, or microbial translocation^5^.

People living with HIV-1 on efficient antiretroviral therapy present with persistent chronic IA^6^. We have previously identified in 120 virologic responders 5 different IA profiles^7^. Among these profiles, Profile 2, was linked to IR. One feature of this profile was an elevated plasma levels of soluble TNF receptor I (sTNFRI). As sTNFRI correlates with TNFα production, and as TNFα induces IR, this is in favor of a causative link between Profile 2 and IR.

Here we probed whether, in a same way one of the IA Profiles we described in a general population could also be linked to IR.

## Results

### A specific immune activation profile is linked to insulin resistance and metabolic syndrome

We recruited 74 women and 76 men with a mean (± SD) age of 61.7 (± 4.3) years. We have previously shown that 5 different IA profiles may be distinguished in this cohort. Profile 1 is characterized by a high proportion of naïve T cells, Profiles 2 and 3 by elevated percentages of terminally differentiated and senescent CD4+ T cells and CD8+ T cells, respectively, Profile 4 by a high proportion of activated NK cells, and Profile 5 by an increase in the percentage of monocytes^5^.

**Figure 1:**
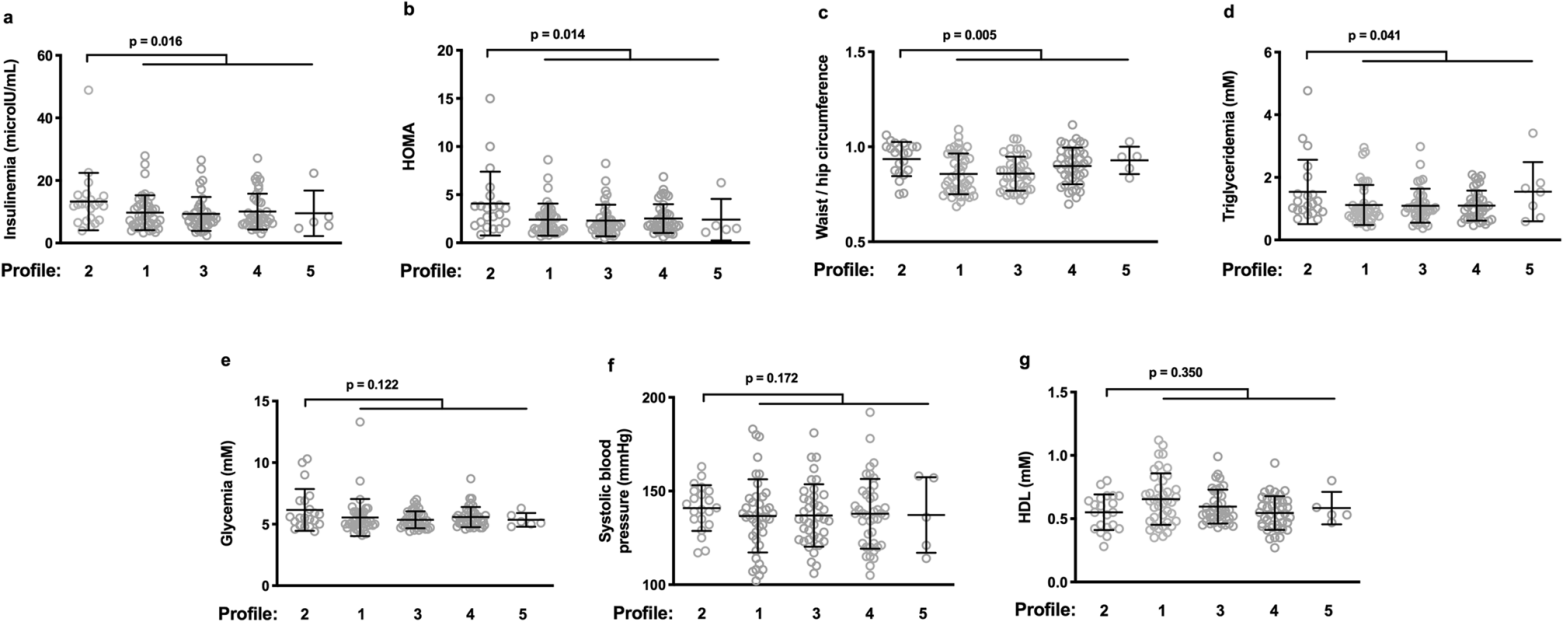
Profile 2 presents with features of insulin resistance and metabolic syndrome. Profile 2 people have higher insulinemia (**a**), HOMA (**b**), waist/hip ratio (**c**), triglyceridemia (**d**), glycemia (**e**), systolic blood pressure (**f**), and HDL (**g**) than the rest of the volunteers.

We tested to see whether one of the IA profiles we had observed in the 150 subjects we have previously analyzed was associated with IR and MetS. The levels of the risk factors defining MetS are given in Table 1 for each IA profile. Compared with the other volunteers, Profile 2 individuals presented higher insulinemia (13.3 ± 9.2 vs 9.7 ± 5.6 μU/mL, *p* = 0.016, Fig. 1a), higher homeostatic model assessment (HOMA) (3.9 ± 3.4 vs 2.4 ± 1.6, *p* = 0.014, Fig. 1b), a larger waist/hip circumference (0.93 ± 0.09 vs 0.87 ± 0.10, *p* = 0.005, Fig. 1c), and higher triglyceridemia (1.5 ± 1.0 vs 1.1 ± 0.6 mM, *p* = 0.041, Fig. 1d). Their glycemia (6.2 ± 1.7 vs 5.5 ± 1.0 mM, *p* = 0.122, Fig. 1e) and systolic blood pressure (141 ± 12 vs 137 ± 18 mmHg, *p* = 0.172, Fig. 1f), were non-significantly higher than the other volunteers, and their HDL (0.55 ± 0.14 vs 0.60 ± 0.16 mM, *p* = 0.350, Fig. 1g) non-significantly lower.

**Table 1:** Patients characteristics.

Characteristic	Variable	All profiles	Profile 1 (N = 43)	Profile 2 (N = 22)	Profile 3 (N = 39)	Profile 4 (N = 41)	Profile 5 (N = 5)
Age	Mean (SD)	61.7 (4.3)	61.4 (4.6)	61.8 (4.8)	62.8 (4.3)	61.2 (3.8)	61.0 (2.1)
**Sex**							
Female	%	48	67	23	59	39	20
Male	%	52	33	77	41	61	80
**Ethnicity**							
African	%	8	10	14	10	2	0
Caucasian	%	92	90	86	90	98	100
Body Mass Index	Mean (SD)	26.6 (4.6)	25.6 (4.9)	27.3 (5.0)	26.8 (4.0)	27.4 (4.9)	25.8 (4.8)
Waist circumference (cm)	Mean (SD)	88.8 (13.3)	85.5 (14.2)	93.4 (14.0)	87.6 (10.5)	90.6 (13.3)	90.8 (16.3)
Female	Mean (SD)	81.5 (10.8)	78.2 (11.2)	80.6 (9.0)	83.6 (10.9)	83.4 (10.8)	95.0
Male	Mean (SD)	95.5 (11.5)	94.5 (11.8)	95.4 (10.7)	96.9 (13.1)	94.9 (12.3)	91.7 (9.8)
Hip circumference (cm)	Mean (SD)	100.5 (8.6)	99.5 (8.6)	99.6 (8.7)	102.2 (8.3)	100.6 (8.6)	97.2 (10.2)
Systolic blood pressure (mmHg)	Mean (SD)	138 (17)	137 (20)	141 (12)	137 (17)	138 (19)	137 (20)
Diastolic blood pressure (mmHg)	Mean (SD)	83 (10)	81 (11)	84 (8)	83 (10)	82 (10)	84 (9)
Glycemia (mM)	Mean (SD)	5.58 (1.17)	5.54 (1.51)	6.16 (1.70)	5.36 (0.69)	5.56 (0.82)	5.40 (0.60)
Cholesterol (mM)	Mean (SD)	2.24 (0.45)	2.22 (0.36)	2.26 (0.55)	2.20 (0.53)	2.25 (0.42)	2.31 (0.42)
HDL (mM)	Mean (SD)	0.59 (0.16)	0.65 (0.20)	0.55 (0.14)	0.60 (0.13)	0.55 (0.13)	0.58 (0.13)
LDL (mM)	Mean (SD)	1.42 (0.38)	1.36 (0.30)	1.40 (0.45)	1.40 (0.43)	1.49 (0.39)	1.49 (0.38)
Triglyceridemia (mM)	Mean (SD)	1.19 (0.68)	1.12 (0.64)	1.54 (1.03)	1.09 (0.54)	1.15 (0.59)	1.26 (0.57)
Insulinemia (μU/mL)	Mean (SD)	10.2 (6.3)	9.7 (5.6)	13.3 (9.2)	9.3 (5.4)	10.1 (5.7)	9.5 (7.3)
Antidiabetic therapy	Number (%)	9 (6%)	4 (9%)	3 (14%)	1 (3%)	1 (2%)	0 (0%)
Antihyperlipidemic therapy	Number (%)	31 (21%)	6 (14%)	8 (36%)	10 (26%)	6 (15%)	0 (0%)
High blood pressure therapy	Number (%)	36 (24%)	8 (19%)	7 (32%)	10 (26%)	8 (19%)	0 (0%)

In line with these biological data, Profile 2 volunteers were more often treated with antihyperlipidemic drugs than the other volunteers (36.4 vs 17.2%, *p* = 0.038), as shown in Table 1. Antidiabetic (13.6 vs 4.7%, *p* = 0.128) and antihypertensive drugs (31.8 vs 20.3%, *p* = 0.229) were non-significantly more often prescribed to Profile 2 individuals than to individuals with another profile.

### Characterization of the immune activation profile linked to insulin resistance

**Figure 2:**
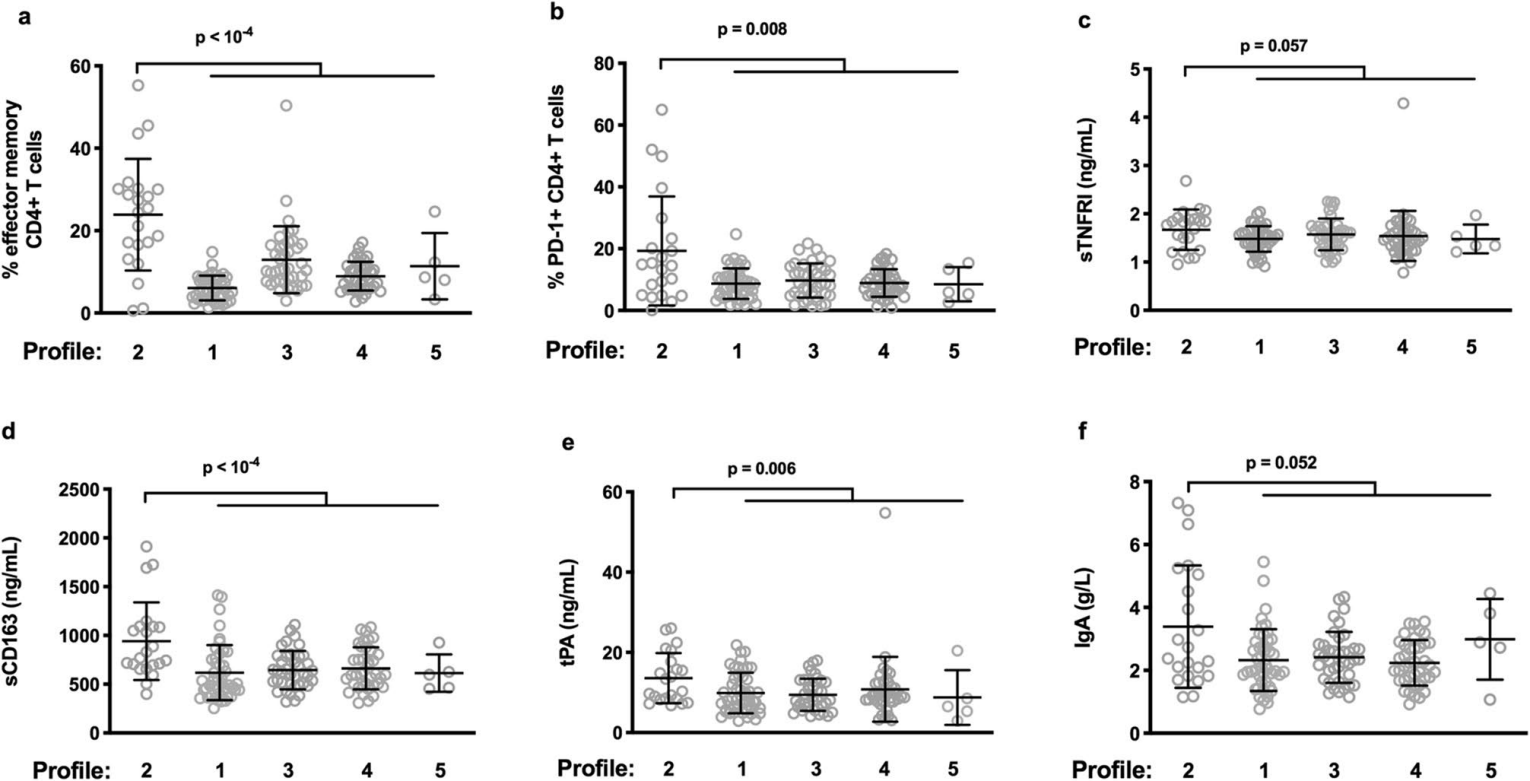
Immune characteristics of Profile 2. Differences between the level of various activation markers in Profile 2 and the other profiles (**a**–**f**).

As Profile 2 is associated with IR and MetS markers, we further analyzed the characteristics of this profile. Consistently with their high proportions of circulating terminally differentiated and senescent CD4+ T cells, people with Profile 2 presented with high proportions of effector memory (24 ± 13 vs 9 ± 6%, *p* < 10^–4^, Fig. 2a) and exhausted (19 ± 18 vs 9 ± 5%, *p* = 0.008, Fig. 2b) CD4+ T cells than the other participants. In addition, their plasma levels of sTNFRI (1.7 ± 0.4 vs 1.5 ± 0.4 mg/L, *p* = 0.057, Fig. 2c), soluble CD163 (sCD163), a marker of monocyte activation (940 ± 398 vs 639 ± 234 ng/mL, *p* < 10^–4^, Fig. 2d), tissue Plasminogen Activator (tPA), a marker of endothelial activation (14 ± 6 vs 10 ± 6 ng/mL, *p* = 0.006, Fig. 2e), and immunoglobulin (Ig)A (3.4 ± 1.9% vs 2.3 ± 0.9 g/L, *p* = 0.052, Fig. 2f), were, or tended to be, also higher than those of the other individuals.

**Figure 3:**
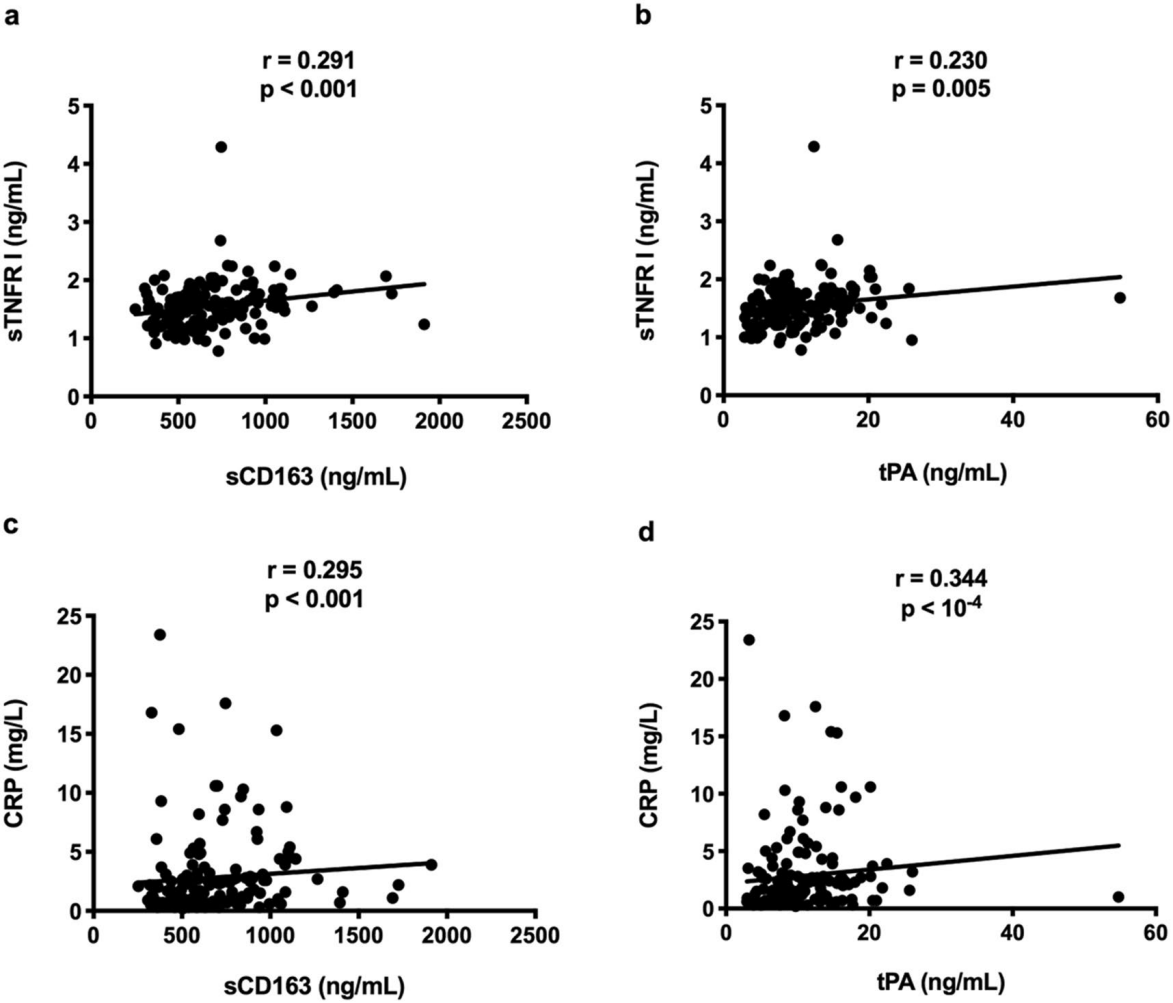
Links between various activation markers in the whole population.

In the hierarchical clustering we have previously performed on all the IA markers, sCD163, tPA, sTNFRI, and IgA were close^5^. Accordingly, in the 150 volunteers, sCD163 (r = 0.291, *p* < 0.001, Fig. 3a) and tPA (r = 0.230, *p* = 0.005, Fig. 3b) levels correlated with sTNFRI plasma concentrations. sCD163 (r = 0.295, *p* < 0.001, Fig. 3c) and tPA (r = 0.344, *p* < 10^–4^, Fig. 3d) also correlated with C-reactive protein (CRP).

### Biomarker selection for Profile 2 signature

Interestingly, using only three markers, the percentage of senescent (CD57+) CD4+ T cells, the circulating levels of IgA and sCD163, we were able to determine whether a volunteer presented or not with Profile 2 with an error rate of 5%, a specificity of 99%, and a sensibility of 77%.

### The IA profiles linked to insulin resistance in HIV-positive individuals and in the general populations have similarities

**Figure 4:**
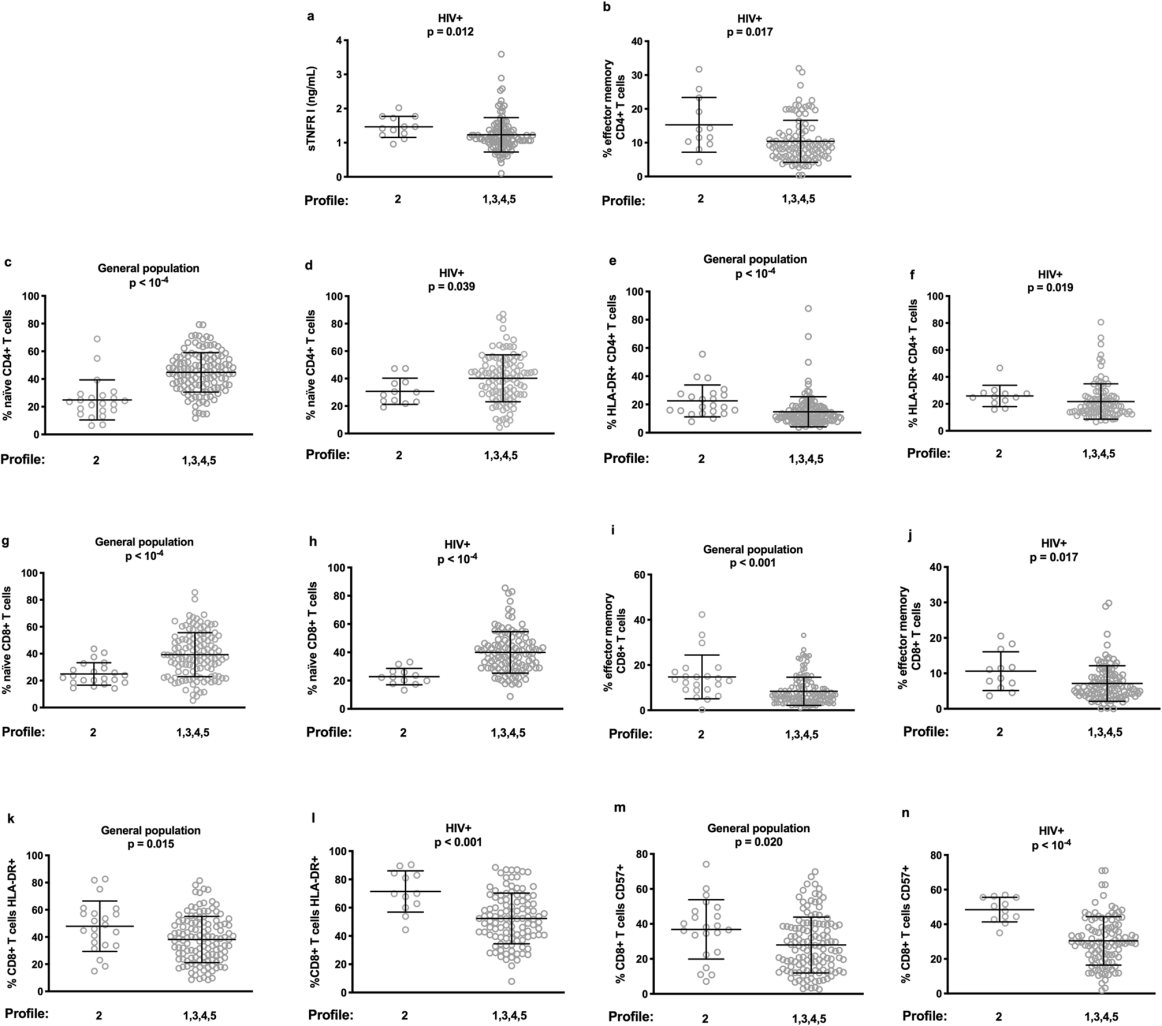
Common immunological characteristics between the general population Profile 2 and the HIV patients Profile 2. The expression of each marker in Profile 2 is compared with its expression in the other profiles.

We tested whether the IR-associated IA profile we have just unveiled in the general population presented common traits with the IR-associated IA profile we have previously identified in people living with HIV-1^7^. Indeed, as compared with the other profiles, HIV-positive Profile 2 was also characterized by high levels of sTNFRI (1.46 ± 0.31 vs 1.23 ± 0.50 ng/mL, *p* = 0.012, Fig. 4a) and high percentages of effector memory CD4+ T cells (15.3 ± 8.1 vs 10.4 ± 6.2%, *p* = 0.017, Fig. 4b). Both Profiles 2 presented a low proportion of naïve CD4+ T cells (24.9 ± 14.4 vs 44.8 ± 14.2%, *p* < 10^–4^, Fig. 4c and 30.7 ± 9.6 vs 40.2 ± 17.1%, *p* = 0.039, Fig. 4d in the general population and in HIV-positive adults, respectively) and a high proportion of activated, HLA-DR-positive, CD4+ T cells (22.5 ± 11.3 vs 14.8 ± 10.7%, *p* < 10^–4^, Fig. 4e and 25.9 ± 7.9 vs 21.7 ± 13.1%, *p* = 0.019, Fig. 4f, respectively). Likewise, in these Profiles 2 there weas a low percentage of naïve CD8+ T cells (24.9 ± 8.3 vs 39.3 ± 16.3%, *p* < 10^–4^, Fig. 4g and 22.8 ± 5.8 vs 40.0 ± 14.7%, *p* < 10^–4^, Fig. 4h, respectively) contrasting with a high percentage of effector memory CD8+ T cells (14.7 ± 9.7 vs 8.4 ± 6.2%, *p* < 10^–3^, Fig. 4i and 10.6 ± 5.5 vs 7.1 ± 5.0%, *p* = 0.017, Fig. 4j, respectively). Finally, two common characteristics of Profiles 2 were high proportions of HLA-DR-positive, CD8+ T cells (47.9 ± 18.5 vs 38.1 ± 17.0%, *p* = 0.015, Fig. 4k and 71.4 ± 14.6 vs 52.4 ± 17.9%, *p* < 10^–3^, Fig. 4l, respectively) and of senescent, CD57+, CD8+ T cells (36.9 ± 17.0 vs 27.9 ± 16.0%, *p* = 0.020, Fig. 4m and 48.5 ± 7.1 vs 30.4 ± 14.0%, *p* < 10^–4^, Fig. 4n, respectively).

### Correlations between immune activation and Metabolic Syndrome markers

**Figure 5:**
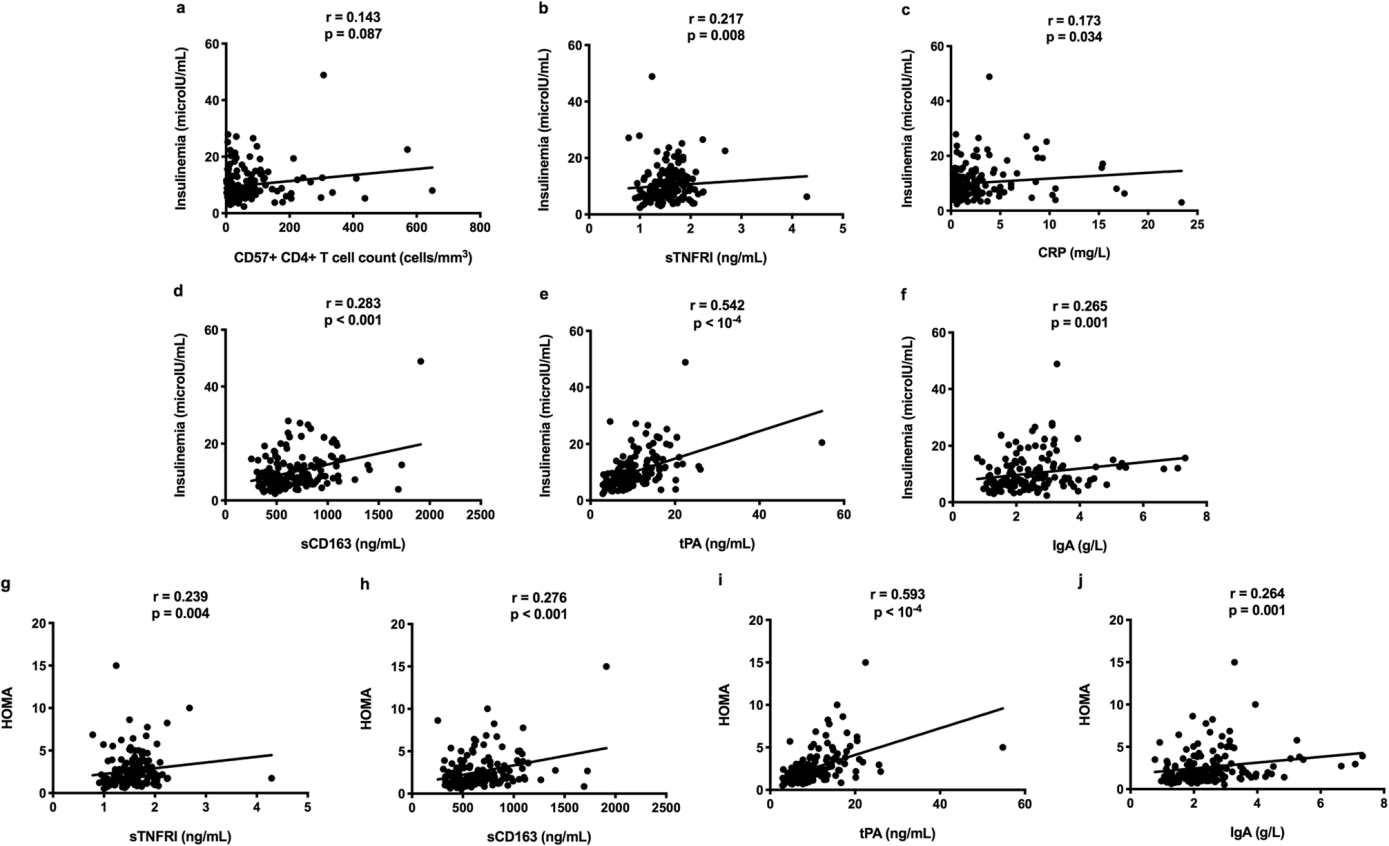
Links between various activation markers, insulinemia and HOMA in the whole population.

Next, we sought links between the characteristics of Profile 2 and markers of IR in the whole population. As a relationship between CD4+ T cell senescence and IR has been recently reported^8^, we looked for such a link in our cohort. We found that the number of senescent CD4+ T cells tended to correlate with insulinemia (r = 0.143, *p* = 0.087, Fig. 5a). In addition, plasma levels of insulinemia correlated with those of sTNFRI (r = 0.217, *p* = 0.008, Fig. 5b) and of another inflammation marker, CRP (r = 0.173, p = 0.034, Fig. 5c). Moreover, insulin concentrations were also strongly linked to the other soluble markers overexpressed in IA Profile 2, sCD163 (r = 0.283, *p* < 0.001, Fig. 5d), tPA (r = 0.542, *p* < 10^–4^, Fig. 5e), and IgA (r = 0.265, *p* < 0.001, Fig. 5f). The same was true for HOMA that correlated with sTNRF1 (r = 0.239, *p* = 0.004, Fig. 5g), sCD163 (r = 0.276, *p* < 0.001, Fig. 5h), tPA (r = 0.593, *p* < 10^–4^, Fig. 5i), and IgA (r = 0.264, *p* = 0.001, Fig. 5j).

**Figure 6:**
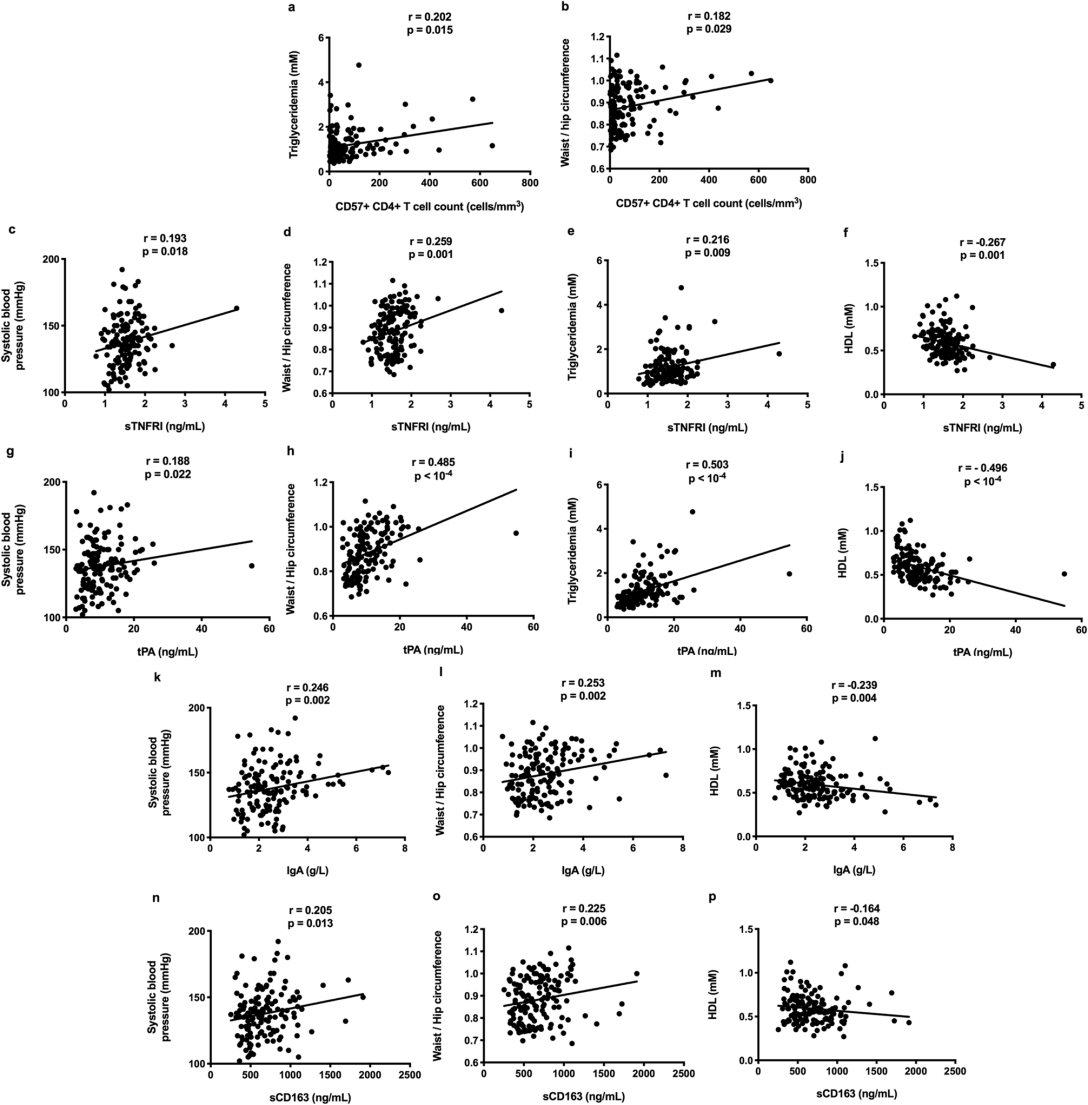
Links between various activation markers and markers of metabolic syndrome in the whole population.

We also looked for links between increased markers in Profile 2 and markers of MetS. Senescent CD4+ T cell count was linked to triglyceridemia (r = 0.202, *p* = 0.015, Fig. 6a) and waist/hip ratio (r = 0.182, *p* = 0.029, Fig. 6b). sTNFRI and tPA correlated with blood pressure, waist/hip ratio, triglyceridemia, and HDL levels (Fig. 6c–j). IgA and sCD163 correlated with blood pressure, waist/hip ratio, and HDL levels (Fig. 6k–p).

### IA Profile 2 is linked to high γGT levels

**Figure 7:**
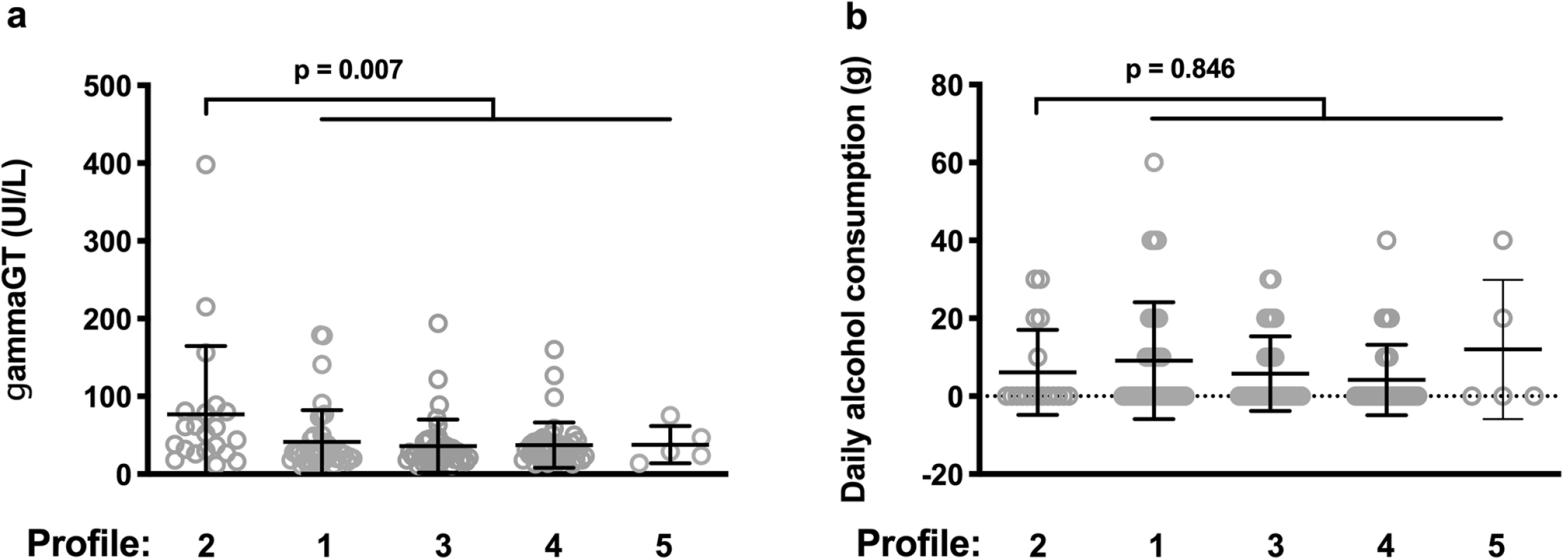
Profile 2 participants present with high yGT levels (**a**) but similar alcohol use (**b**) as compared with the other participants.

One of the consequences of MetS is liver steatosis^2^. Liver steatosis is the most common cause of elevated gamma-glutamyltransferase (γGT)^9^. To test the hypothesis that persons with IA Profile 2 might develop more frequently this disease, we compared γGT levels in the persons who volunteered for this study according to their IA profile. Individuals with IA Profile 2 presented with higher levels of γGT than the other individuals (76 ± 88 vs 38 ± 34 UI/L, *p* = 0.007, Fig. 7a). Another potential cause of elevated serum γGT levels is alcohol consumption, but there was no difference in alcohol use between participants with Profile 2 and the other participants (6.1 ± 10.9 vs 8.2 ± 22.1 g, *p* = 0.846, Fig. 7b).

## Discussion

In this study, we have shown that one IA profile is clearly linked to some markers of IR and MetS, eventhough subjects with this profile received antidiabetic, antihyperlipidemic and antihypertensive therapies more often than the other subjects. This is phenocopying our report of a link between one IA profile and hyperinsulinemia as well as hypertriglyceridemia in HIV patients^7^. Of note, both IR-linked IA profiles, in people living with HIV-1 and in a general population, present many common immunological characteristics. This is also in line with the correlation between adipose tissue inflammation and IR reported by many studies^10^. In particular, macrophage activation, TNFα overproduction^11^, and CD163 shedding^12^ have been observed in human obese adipose tissue. Even activated B cells have been shown in this tissue^13^.

Strikingly, in this present work we found that the IA profile linked to insulin resistance was characterized by a high level of sTNFRI, a finding common to our observation in people living with HIV^7^. As sTNFRI is a marker of TNFα production, and as TNFα inhibits insulin signaling^4^, both findings argue for a causative link between IA Profile 2 and insulin resistance.

We also observed that hyperinsulinemia was linked to the levels of circulating sCD163. In the course of inflammation, CD163 is shed from the surface of monocytes and macrophages by the metalloproteinase TNFα-converting enzyme (TACE), which is also responsible for the release of TNFα from the surface of immune cells^14^. Therefore, sCD163 is considered as a marker of monocyte and macrophage activity and of TNFα production. Indeed, TNFα production and sCD163 levels have previously been shown to be related^15^ as we observed here (Fig. 3a). White adipose tissue (WAT) inflammation and IR are inter-related^16^. This WAT inflammation results in CD163 mRNA overexpression in WAT macrophages^17^ which correlates with sCD163 blood concentrations^12^. Accordingly, sCD163 levels have been reported to be linked to adiposity^18–22^ on one hand and to IR^15,20,21,23–25^ on the other hand. sCD163 has even be identified as a predictive marker of type 2 diabetes in the general population independently of age or BMI^23^. Thus, the fact that sCD163 levels in Profile 2 are higher than in the other profiles may be considered as an additional argument for a causative link between Profile 2 and IR.

tPA is released by activated endothelial cells functioning both as a serine protease favoring thrombolysis by converting plasminogen to plasmin and as an inflammatory cytokine^26^. tPA activates NFκB, inducing inflammatory cytokines production, and modulates inflammatory infiltration, particularly macrophage migration, in various organs^26–30^. In line with these proinflammatory properties, tPA levels have been linked to CRP and fibrinogen levels in another study^31^, and to sTNFRI as well as to CRP in the present study (Fig. 3b,d). These characteristics might explain why tPA is correlated with IR and predictive for the development of type 2 diabetes^32–34^. Thus, the high level of tPA in Profile 2 may be interpreted as a third argument for a causative link between Profile 2 and IR.

In addition to monocyte/macrophage and T cell activation, B cell overactivity has been observed in the visceral adipose tissue (VAT) of obese mice^35,36^ and men^13^. In VAT, B cell activation induces the production of IFNγ and other inflammatory cytokines by T cells, fueling IR^36^. This aligns with the fact that B cell deficient mice on a high fat diet show better insulin sensitivity than wild-type mice^36^. One consequence of this B cell activation might be an increase in serum IgA levels which has been linked to obesity^37,38^, MetS^37,38^, and diabetes^37^, as we observed here in Profile 2.

Globally, these data lead to a model where IA Profile 2 favors IR. Yet, it may be argued that conversely, IR may favor Profile 2. First, IR is responsible for an increase in circulating free fatty acids able to activate hepatic macrophages and thereby to increase the level of sCD163^39^. In addition, IR and hyperglycemia cause oxidative stress, TACE activation, and CD163 shedding^14^. Second, a positive causal effect of IR on tPA has been revealed via a Mendelian randomization analysis^32^. This causal effect could be mediated by a defect in endothelium-derived nitric oxide^40^. Third, hyperproduction of IgA might also be a consequence of metabolic disorders, as suggested by the report that treatment of morbid obesity by adjustable gastric banding leads to reduction in IgA values^41^.

Thus, the links between Profile 2 and IR might be bidirectional, resulting in a vicious circle. Whatever the nature of these links, it would be interesting to test whether Profile 2 is predictive for the establishment of MetS and for the morbidities it fuels. The combination of activation markers characterizing Profile 2 might then provide us with a predictive signature. The possibility of reducing the size of this signature to three markers adapts immune profiling to routine. Last, but not least, deciphering the soluble factors which are overproduced by Profile 2 individuals, and which may cause IR might uncover pathways to pharmacologically target and prevent MetS.

One of the limitation of our work is that our study population is not representative of the general population. We recruited adults who volunteered for a free health checkup. Therefore, the majority of these volunteers were in a precarious socio-economical situation, and overweight. Another limitation is that the present study is cross-sectional, highlighting only correlations. Further analysis is needed to establish whether there are causative links between some types of immune activation and IR.

Globally, we show that one of the IA profiles observed in a general population is linked to IR, MetS, and possibly to one consequence of MetS, liver steatosis. Our findings open the way to the identification of markers predictive for metabolic syndrome and the morbidities it drives, and of new causative factors.

## Methods

### Study design

We have previously described the characteristics of the 150 volunteers we enrolled for this study^5^. Pregnant or breastfeeding women, people under immunomodulatory treatment, with cancer, acute infection, autoimmune or autoinflammatory disease were not included. Fifty-nine percent of these volunteers were in a precarious socio-economical situation. Our protocol was performed in accordance with the relevant guidelines and regulations and approved by the French Ethics Committee Sud Est IV. All patients had provided written informed consent. The trial was registered on ClinicalTrials.gov under the reference NCT04028882.

### Metabolic and immunologic markers in peripheral blood

Blood was collected after eight hours of fasting on EDTA Vacutainer tubes (Becton Dickinson, Le Pont-de-Claix, France), immediately centrifuged (delay < 4 h) and plasma was frozen at -80 °C on several aliquots until analysis. Insulinemia were measured using electrochemiluminescence immunoassay “ECLIA” on Cobas e602 analyzer from Roche (Roche diagnostic, Meylan, France) using Roche reagent kits and calibrators. Insulinemia was determined from plasma aliquot never thawed. Lower detection limit was 0.2 µU/mL (1.39 pmol/L) and intra-and inter-assay was < 1.5% and < 5%, respectively. Blood triglycerides, HDL, LDL, and total cholesterol levels were quantified on the Cobas8000/e502© analyzer from serum collected into SST II Vacutainer tubes (Becton Dickinson). The determination of the other markers has been previously described^5^.

### Statistical analysis

We used t-test or Mann–Whitney test to compare markers and IA profiles. The links between markers were determined by Spearman rank correlations. Fisher test or χ^2^ test was used to compare qualitative covariates. 

The R software version R 3.5.1 (July, 2018) was used to perform the analysis described in this section. To select an optimal number of variables and create a parsimonious predictive model of the Profile 2, we chose genetic algorithms (GAs)^42,43^. GAs are optimization algorithms, inspired by Charles Darwin’s idea of natural selection. They provide approximate solutions to complex optimization problems. In a first step, a population of potential solutions is randomly generated. Then, this population evolves through the iterative application of mutation, cross-over and selection. The natural selection preferentially preserves the fittest individuals over the successive generations. An evolutionary algorithm improves the selection over time and allows the best solution to emerge from the best of prior solutions.

In our application^44,45^, solutions are subsets (combinations) of the immunological markers (the features) mentioned above. Specifically, the mutation randomly alters a solution by feature addition, removal or substitution. The cross-over randomly combines the features of two solutions. Selection is the only operator increasing the quality of solutions across generations. It relies on a fitness function (to be optimized) quantifying the solution quality.

A Linear Discriminant Analysis (LDA)^46^ is applied on each solution using the R package MASS. To avoid over-fitting a cross-validation is used to evaluate the accuracy. The fitness function uses this accuracy penalized by the subset size to favor parsimonious solutions. In this goal, we also chose 10 as the maximal size for subsets. In order to favor solution robustness, the genetic algorithm was run four times and all solutions of the final generations were evaluated through 30 runs of independent linear discriminant analysis with 2-fold cross validation. Solutions were ranked according to their average correct classification rate during the cross-validation process.
